# Research Progress and Trends in Metabolomics of Fruit Trees

**DOI:** 10.3389/fpls.2022.881856

**Published:** 2022-04-29

**Authors:** Jing Li, Guohua Yan, Xuwei Duan, Kaichun Zhang, Xiaoming Zhang, Yu Zhou, Chuanbao Wu, Xin Zhang, Shengnan Tan, Xin Hua, Jing Wang

**Affiliations:** ^1^Key Laboratory of Saline-Alkali Vegetation Ecology Restoration, Ministry of Education, Northeast Forestry University, Harbin, China; ^2^Institute of Forestry and Pomology, Beijing Academy of Agriculture and Forestry Sciences, Beijing, China; ^3^Key Laboratory of Biology and Genetic Improvement of Horticultural Crops (North China), Ministry of Agriculture and Rural Affairs, Beijing, China; ^4^Beijing Engineering Research Center for Deciduous Fruit Trees, Beijing, China; ^5^Analysis and Test Center, Northeast Forestry University, Harbin, China

**Keywords:** fruit tree, metabolomics, quality, resistance, mQTL, mGWAS

## Abstract

Metabolomics is an indispensable part of modern systems biotechnology, applied in the diseases’ diagnosis, pharmacological mechanism, and quality monitoring of crops, vegetables, fruits, etc. Metabolomics of fruit trees has developed rapidly in recent years, and many important research results have been achieved in combination with transcriptomics, genomics, proteomics, quantitative trait locus (QTL), and genome-wide association study (GWAS). These research results mainly focus on the mechanism of fruit quality formation, metabolite markers of special quality or physiological period, the mechanism of fruit tree’s response to biotic/abiotic stress and environment, and the genetics mechanism of fruit trait. According to different experimental purposes, different metabolomic strategies could be selected, such as targeted metabolomics, non-targeted metabolomics, pseudo-targeted metabolomics, and widely targeted metabolomics. This article presents metabolomics strategies, key techniques in metabolomics, main applications in fruit trees, and prospects for the future. With the improvement of instruments, analysis platforms, and metabolite databases and decrease in the cost of the experiment, metabolomics will prompt the fruit tree research to achieve more breakthrough results.

## Introduction

To comprehensively and quantitatively study key metabolites in a biological system, “metabolomics,” which refers to the qualitative and quantitative analysis of all metabolites with molecular weight < 1,000 Da in organisms or cells, was proposed by Nicholson in 1999 (Nicholson et al., 1999).

Unlike DNA, RNA, and proteins, which consist of several fixed and known structural units, metabolites are reactive, are structurally diverse, and have a wide range of concentrations. Metabolites do not follow a fixed structural module and have different physical properties (Sumner et al., 2003). The Human Metabolome Database (HMDB) has identified more than 40,000 human metabolites. The environment in which plants grow and reproduce is far more complex than those of humans and animals and often disturbed by a variety of biotic and abiotic factors. Considering their own factors, plants can only adjust the whole process of their life cycle in a more flexible and diverse way, with the total number of metabolites produced by plants ranging from 200,000 to 1 million (Dixon and Strack, 2003).

**FIGURE 1:**
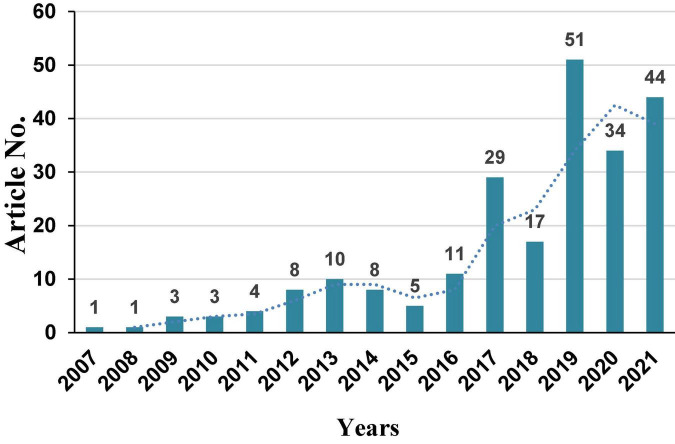
Published research articles about metabolomics of fruit tree (data from Web of Science).

Fruits provide irreplaceable nutrition to human and have an increasingly important impact on human health. Compared with animals and main crops, metabolomics of fruit trees starts later. But fruits are beneficial to human health due to their diverse secondary metabolites, including organic acids, flavonoids, anthocyanins, terpenes, etc., which are very suitable for the study by metabolomics technology. Thus, a rapid increase of research articles appears from 2017 to 2021 (Figure 1) according to the Web of Science^1^. Many important research results have been achieved in metabolomics of a fruit tree in recent years. In this article, main achievements in metabolomics of fruit tree will be introduced.

## Metabolomics Strategies and Key Techniques

### Metabolomics Strategies

Metabolites in plants can generally be divided into primary and secondary metabolites. Primary metabolites are the basic material conditions necessary for the growth and development of organisms, while secondary metabolites are metabolites produced only at a certain stage or period of growth of an organism (Koch, 2004; Carreno-Quintero et al., 2013). Depending on the purpose of the experiment, researchers can choose individual metabolomics *strategies*, including targeted metabolomics, non-targeted metabolomics, pseudo-targeted metabolomics, and widely targeted metabolomics.

Targeted metabolomics assays are performed on known compounds with targeted extraction methods and high-purity standards to quantify the target metabolism. Generally, targeted metabolomics focuses on fewer compounds and can be searched directly in classified databases, for example, databases for glycans and lipids such as LipidMaps and LipidBank (Cajka and Fiehn, 2016). Non-targeted metabolomics assays are performed on unknown compounds. It covers as many compounds of different properties and classes as possible in the extraction and detection, with a relatively broad coverage of substances. However, both the qualitative and quantitative properties of compounds are compromised by the lack of standards. Generally, non-targeted metabolomics studies are used to screen for differential metabolites in different treatments, and targeted metabolomics studies are used for the next more precise study.

To overcome the disadvantages of non-targeted metabolomics, widely targeted metabolomics, also known as second-generation targeted metabolomics, allows for the simultaneous detection of thousands of substances by the establishment of a database of thousands of metabolite specimens (Wei et al., 2013). Pseudo-targeted metabolomics is a quantitative ion selection algorithm based on untargeted metabolomics. It is used to perform quantitative ion selection for all detected metabolites. The ion-pair information of metabolites is obtained by high-resolution mass spectrometry (HR-MS), and the abundance of metabolites is measured by targeted multiple reaction monitoring (MRM). The method has high coverage, good linearity, and reproducibility and does not require standards to limit the metabolites’ detection. Both known and unknown metabolites in the sample can be measured (Zheng et al., 2020).

Spatial metabolomics integrates traditional metabolomics technology and MS imaging technology, which can not only identify what substances are contained in a sample and their contents as traditional metabolomics but also detect the spatial distribution information of compounds in a single experiment to achieve qualitative and quantitative positioning at the same time (Li et al., 2018).

The comparison of advantages and disadvantages of different metabolomic strategies is shown in Table 1.

**TABLE 1:** Different strategies of metabolomics.

Metabolomic	Advantages	Disadvantages	Application
Targeted metabolomics	Qualitative and precise quantitative	Limited to detection of compounds of known molecular weight, low throughput	Qualitative and quantitative detection of a small number of known compounds
Non-targeted metabolomics	High throughput, wide detection range	Qualitative difficulty, quantitative accuracy is low	High throughput detection of known and unknown compounds
Widely-targeted metabolomics	High throughput, accurate qualitative and quantitative	Limited to library-building compounds	Qualitative and quantitative detection of a large number of known compounds
Pseudo-targeted metabolomics	Repeatability, high throughput, wide detection range	/	High throughput detection of known and unknown compounds
Spatial metabolomics	Qualitative, quantitative, positioning, high throughput	/	Qualitative, quantitative, and localized analysis of a large number of known or unknown compounds directly

### Key Techniques in Metabolomics

The exploration of the diversity of fruit tree metabolism and potential molecular mechanisms by which fruit tree cells control their own chemical composition depends on the advances of analytical methods.

#### Sample Processing

Samples should be collected representatively, with priority given to normally developing plants. Furthermore, 3–6 individuals should be selected as a source of biological replicates to avoid differences due to excessive individual differences. Ice boxes or ice packs should be used to collect samples in the field to reduce the degradation of metabolites by enzymes that are still active after sample collection. At the same time, samples should be collected with certain accuracy, and the samples of the experimental and control groups should be consistent in terms of time (Urbanczyk-Wochniak et al., 2005), site, and processing conditions, as far as the experiment allows. If the kernels are not studied, it should be removed with a scalpel in a low-temperature metal bath on an RNAase reagent-treated bench. For the study of the peel, the pulp should be taken as less as possible (Montefiori et al., 2009; de Godoy et al., 2013; Rudell et al., 2017). Freezing of the whole fruit should be avoided if possible during the sampling process, which will make the subsequent freeze-drying and grinding work difficult, and once the whole fruit is freeze-thawed, the whole experimental results will be affected. Then, the sample will be stored in a suitable volume of a centrifuge tube, marked and quickly put into liquid nitrogen for precooling, and then put into −80°C refrigerator for storage. Note that the samples should not be stored in tin foil, self-sealing bags, Ziploc bags, plastic bags, etc.; such packaging is easy to break under low temperature or in the process of transportation and easy to make the markings blurred, resulting in sample confusion.

The metabolites are usually extracted separately with water or organic solvents (e.g., methanol, chloroform, etc.) to obtain aqueous and organic solvent extracts, thus separating the non-polar lipophilic phase from the polar phase. To enrich the concentration of metabolites during the study, the process of vacuum concentration and spin-drying of the extracts before redissolution is often added. In the process of sample analysis method establishment, the optimal method can be figured out by trying different combination of extraction solvents, extraction temperatures, ratios of sample and solvent, redissolved solvent, mobile phase gradients, and mobile phases and by the controlled variable method (Kim and Verpoorte, 2010). The detection of non-targeted metabolites should include as many compound peaks as possible, and the detection of targeted metabolites should ensure that all concerned metabolites can be detected. The sample sequence requires the insertion of quality control samples at intervals of 10 or 20 during the detection by liquid chromatography-MS (LC-MS), which generally includes all compounds of the sample being tested or a mixture of a representative batch of samples. In addition, compounds not contained in the fruit trees are added as internal standards during the extraction of the samples to calibrate the loss of sample compounds during the extraction and detection process and thus to correct the peak areas of other compounds. *In vivo* direct-immersion solid-phase microextraction followed by gas chromatography-time-of-flight mass spectrometry method (SPME GC × GC-TOF-MS) can detect volatile compounds directly without extracting the compounds (Risticevic et al., 2016, 2020).

**FIGURE 2:**
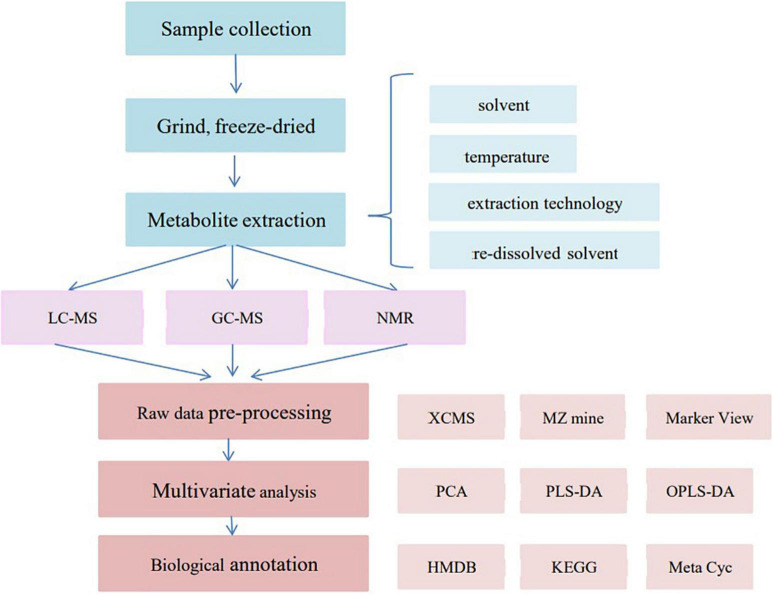
Metabolomics sample preparation, analysis, and data processing flowchart.

The flowchart of metabolomics sample preparation, analysis, and data processing is shown in Figure 2.

#### Analysis

Chromatography is used for the purpose of separating metabolites according to the different affinities of compounds and columns and is generally divided into LC and gas chromatography (GC). LC is used to detect non-volatile compounds, polar compounds, thermally unstable compounds, and high-molecular-weight compounds (including proteins, peptides, and polymers). The mobile phase generally uses methanol, acetonitrile, water, or isopropanol; the added acid is generally acetic acid or formic acid, inorganic acid cannot be used (inorganic acid may corrode the pipeline); the added base is generally ammonium hydroxide or ammonia, generally does not use metal bases (will corrode the pipeline). Trifluoroacetic acid (TFA) helps to improve the separation of the liquid phase but will produce ion suppression in the positive and negative ion modes of MS. Triethylamine/trimethylamine (TEA/TMA) contributes to the formation of negative ions. GC can be used for the detection of volatile compounds, the mobile phase is an inert gas, and samples with a certain vapor pressure and good stability at column temperature can be detected. Substances with low volatility and easily decomposed by heat can be transformed by derivatization into derivatives with high volatility and good thermal stability for detection. *N*-methyl-*N*-trimethylsilyl trifluoroacetamide (MSTFA) and methoxamine hydrochloride are often used as derivatization reagents, as preliminary studies have shown that these compounds are the most suitable for the derivatization of plant metabolites (Fiehn et al., 2000).

Mass spectrometry separates some of the ions based on the mass to charge ratio. The ions pass through the analyzer and are separated according to different mass to charge ratios. Ions with the same mass to charge ratio are clustered together to form a mass spectrogram. Commonly used mass spectrometers are triple quadrupole mass analyzer (Yost and Enke, 1978), time-of-flight mass analyzer, ion trap mass analyzer, and electrostatic field orbital trap. The triple quadrupole mass analyzer consists of four straight rod electrodes suspended equidistantly parallel to the axis. Under the action of a certain DC/VC, ions with m/z meeting certain requirements can pass through the quadrupole to the detector, while other ions are filtered out. The advantages of the triple quadrupole are light weight, small size, and low cost. The time-of-flight mass analyzer uses pulses to extract ions from an ion source. The ions are accelerated by an accelerating voltage, have the same kinetic energy, and enter the drift tube. Ions with a small mass to charge ratio are the fastest and reach the detector first. Larger ions reach the detector last. The advantages of TOF MS are a wide range of mass to charge ratio of the detected ions, high sensitivity, and suitability for secondary tandem MS. A fast scanning speed is suitable for studying very fast processes. The principle of an ion trap mass analyzer is similar to that of quadrupole mass analyzer. When the HF voltage amplitude and HF voltage frequency are fixed to a certain value, only ions with a certain mass to charge ratio can be stabilized on a certain track in the trap. By rotating and changing the electrode voltage, different m/z ions fly out of the trap and reach the detector. The advantage of ion trap mass spectrometer is that a single ion trap can realize multistage “time” tandem MS, simple structure, low price, high cost performance, high sensitivity, and large mass range. The electrostatic field ion orbitrap is where analytes are ionized in an ion source and then sequentially enter a quadrupole, a C-trap, and an Orbitrap. The electrostatic field ion orbitrap mass spectrometer can perform high precision mass scans, and it is more stable than other mass analyzers.

Mass spectrometry characterizes compounds by different characteristic ions of different compounds. Chromatographic methods enhance the coverage of metabolites and improve the quantitative accuracy of MS. The most commonly used separation and analysis methods are LC-MS (Vos et al., 2007), GC-MS (Lisec et al., 2015). For LC-MS and GC-MS methods, internal standards are added before sample processing to correct errors caused by sample pretreatment and instrument response.

#### Data Processing

Data processing in metabolomics generally consists of two major steps, namely, data preprocessing and statistical analysis (Ma and Qi, 2021). Data preprocessing includes steps such as removing system noise signals, removing disturbing signals caused by system instability, and removing operational errors to provide a more reliable dataset for the next statistical analysis. Prior to formal data processing, tools such as XCMS, MZmine, and MarkerView are used to process the original peak area and other data for non-targeted metabolomics data analysis. The first step of the statistical analysis is unsupervised multivariate statistical analysis, usually using the principal component analysis (PCA). The second step may be the univariate statistical analysis to screen for variables with statistically significant differences in different groups. The third step is a supervised multivariate statistical analysis such as partial least squares discriminant analysis (PLS-DA) to select the variables that contribute more to the classification of the sample, i.e., screen for markers.

The purpose of the PCA analysis (Shaffer, 2002) is to find specific values that produce differences in a large amount of sample data. PLS-DA analysis will classify samples into groups, which is good for finding the similarities and differences between sample groups; orthogonal partial least squares discriminant analysis (OPLS-DA) can also distinguish the group differences between samples, but this analysis can better focus on some positive correlation data. The results of data analysis are also subject to the *t*-test and variable importance in projection (VIP) ranking values to screen for differential metabolites. It is generally accepted that variables meeting both *P* < 0.05 and VIP > 1.0 are differential metabolites. Metabolic pathways can be resolved using databases such as HMDB, KEGG, Reactome, BioCyc, and MetaCyc.

## Application of Metabolomics in Fruit Trees

**FIGURE 3:**
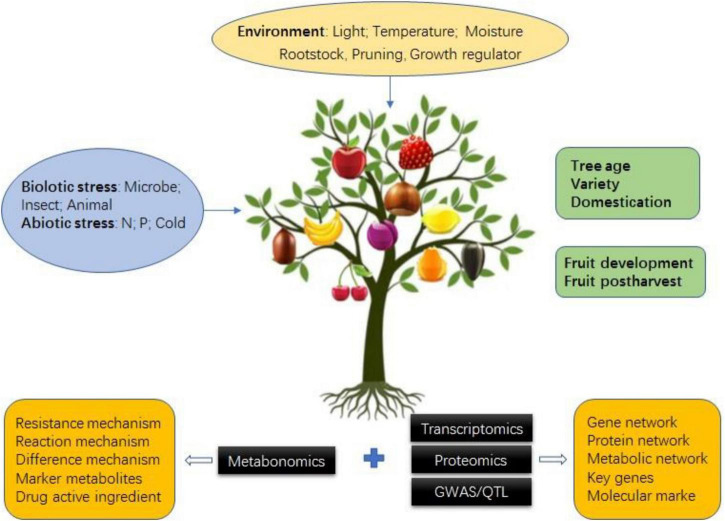
Application of metabolomics with/without multi-omics in fruit tree.

Fruit tree metabolomics has developed rapidly in recent years, and many important research results have been achieved in combination with transcriptomics, genomics, proteomics, quantitative trait locus (QTL), and genome-wide association study (GWAS) (Tables 2, 3). These research results mainly focus on the mechanism of fruit quality formation, metabolite markers of special quality or physiological period, key genes and metabolites of fruit tree resistance to biotic/abiotic stress, the influence of environment on the fruit tree, and fruit tree population genetic basis by combining with QTL and GWAS to narrow down QTL regions, predict candidate genes, construct the regulatory network, etc. (Figure 3).

**TABLE 2:** Application of metabolomics and multi-omics in fruit tree.

Species	Tissue	Muti-omics^a^	Strategies of metabolomics	Technology of metabolomics	Metabolic	References
Apricot (*Prunus armeniaca* L.)	Pulp	T + M	Targeted	UHPLC-APCI-MS/MS	Carotenoid	Zhou et al., 2021
Pineapple (*Ananas comosus* L.)	Pulp	T + M	Widely targeted	UPLC-MS/MS GC-MS	Fatty acid	Hong et al., 2021
Kiwi fruit (*Actinidia chinensis*)	Pulp	T + M	Widely targeted	GC-MS HS-SPME-GC-MS	Sugars, organic acids, volatiles	Wang et al., 2022
Longan (*Dimocarpus longan* Lour.)	Pericarp	T + M	Widely targeted	HPLC UPLC-MS/MS	Anthocyanins	Yi et al., 2021
Tomato (*Solanum lycopersicum*)	Pulp	T + M	Targeted	GC-MS	Amino acid and isoprenoid amino acid	Dominguez et al., 2021
Sweet Cherry (*Prunus avium* L.)	Pulp	T + M	Untargeted	UHPLC-MS/MS	Flavonoids	Berni et al., 2021
Peach (*Prunus persica* L.Batsch)	Pulp	T + M	Untargeted	GC × GC-ToF-MS	Volatile organic compounds	Muto et al., 2020
Pear (*Pyrus bretschneideri* × *Pyrus pyrifolia*)	Pulp	T + M	Untargeted	LC-MS	Gibberellin GA3	Wang et al., 2021f
Apple (*Malus* × *domestica* Borkh)	Pulp	T + M + P	Widely Targeted	UPLC-QQQ-MS	Carbohydrates, flavonoids, amino acids	Wang et al., 2021a
Strawberry (*Fragaria* × *ananassa*)	Pulp	T + M	Untargeted	GC-TOF-MS	Organic acids and sugars	Vallarino et al., 2019
Apple (*Malus* × *domestica* Borkh)	Pulp and pericarp	T + M	Untargeted	LC-Q-TOF-MS	Phenol	Khan et al., 2012b
Jujube (*Ziziphus jujuba* Mill.)	Pulp	T + M	Widely targeted	LC-MS/MS	Triterpenoids, alkaloids, and flavonoids	Zhang et al., 2021
Jujube (*Ziziphus jujuba* Mill.)	Pericarp	T + M	Targeted		Flavonoid and anthocyanins	Zhang et al., 2020
Citrus (*Citrus reticulata*)	Pericarp	T + M	Untargeted	GC-MS	Sugars, organic acids, amino acid	Lu et al., 2017
Fig (*Ficus carica* L.)	Pericarp	T + M	Untargeted	LC-MS	Flavonoid and anthocyanins	Wang et al., 2017
Apple (*Malus* × *domestica* Borkh)	Pulp and pericarp	T + M	Untargeted	UPLC-TOF-MS	Flavonoid	Wang et al., 2021c
Apple (*Malus* × *domestica* Borkh)	Root and leaf	T + M	Untargeted	LC-ESI-MS/MS	Flavonoid	Sun et al., 2021
Citrus (*Citrus reticulata*)	Pericarp	T + M	Untargeted	HS-SPME-GC–MS GC-MS	Sugars, organic acids, volatiles	Tang et al., 2018
*Cerasus humilis* (Bge.) Sok	Pericarp	T + M	Untargeted	UPLC-MS/MS	Anthocyanins	Ji et al., 2020
Apple (*Malus* × *domestica* Borkh)	Pulp and pericarp	T + M	Widely targeted	LC-MS/MS GC-MS	Sugars, organic acids, amino acid	Xu et al., 2020
Citrus (*Citrus reticulata*)	Root	T + M + P	Untargeted	NMR	Polar component	Padhi et al., 2019
Walnut (*Juglans regia* L.)	Pulp and pericarp	T + M + P	Untargeted	LC-MS	Raffinose	Wang et al., 2021b

*^a^T is transcriptomics; M is metabolomics; P is proteomics.*

**TABLE 3:** Application of metabolomics in fruit tree.

Species	Tissue	Strategies of metabolomics	Technology of metabolomics	Application	References
Pear (*Pyrus bretschneideri* × *Pyrus pyrifolia*)	Flower buds	Untargeted	GC-TOF-MS	Environment	Horikoshi et al., 2018
Blueberry (*Vaccinium* spp.)	Flower buds	Untargeted	GC-TOFMS	Abiotic stress	Wang et al., 2021d
Peach (*Prunus persica* L.Batsch)	Flower buds	Widely targeted	LC-MS	Biotic stress	Zhang et al., 2019
Pear (*Pyrus bretschneideri* × *Pyrus pyrifolia*)	Stem	Untargeted	LC-MS-MS	Environment	Cui et al., 2021
Banana (*Musa nana* Lour.)	Pulp	Untargeted	GC–MS	Environment	Nascimento et al., 2019
*Juniperus communis* L.	Pulp	Untargeted	^1^H NMR	Biotic stress	Falasca et al., 2014
African baobab fruit (*Adansonia digitata* L.)	Pulp	Untargeted	UHPLC-HRMS/MS + HS-SPME/GC-MS GC-MS	Quality	Baky et al., 2021
Ginseng (*Panax ginseng Meyer*)	Pulp	Untargeted	GC-MS	Quality	Park et al., 2019
Olive (*Olea europaea* L.)	Pulp	Untargeted	LC-MS + GC-MS	Quality	Olmo-García et al., 2018
Dates (*Phoenix dactylifera* L.)	Pulp and pericarp	Untargeted	SPME GC–MS	Quality	Khalil et al., 2017
Apple (*Malus* × *domestica* Borkh.)	Pulp and pericarp	Untargeted	DI-SPME-GCxGC-ToFMS	Quality	Risticevic et al., 2016, 2020
Strawberry (*Fragaria* × *ananassa*)	Pulp and pericarp	Untargeted	(MALDI-TOF IMS)	Quality	Wang et al., 2021e
Citrus (*Citrus reticulata*)	Pulp and pericarp	Untargeted	LC-MS	Quality	Wang et al., 2016
Citrus (*Citrus reticulata*)	Pericarp	Untargeted	UPLC-QQQ-MS	Biotic stress	Wang et al., 2020
Citrus (*Citrus reticulata*)	Leaf	Targeted	LC-MS/MS	Biotic stress	Suh et al., 2021
Grapevine (*Vitis vinifera* L.)	Pulp and pericarp	Targeted	UHPLC	Genetic mechanism	Arrizabalaga-Arriazu et al., 2021
Grapevine (*Vitis vinifera* L.)	Stem	Widely targeted	LC-HRMS	Genetic mechanism	Teh et al., 2019
Mulberry (*Morus alba* L.)	Leaf and phloem sap	Untargeted	GC-MS	Biotic stress	Gai et al., 2014
Citrus (*Citrus reticulata*)	Pericarp	Untargeted	HPLC-PDA-QTOF-MS	Biotic stress	Ballester et al., 2013
Apple (*Malus* × *domestica* Borkh)	Immature fruit and shoot	Untargeted	LC-MS	Biotic stress	Klee et al., 2019
Genipap (Genipa americana L.)	Fruit	Untargeted	UHPLC-MS	Quality	Dickson et al., 2020
Rambutan (*Nephelium lappaceum* L.)	Seed	Untargeted	UPLC-qTOF-MS/MS	Quality	Lee et al., 2020
Crescentia cujete (*Bignoniaceae*)	Fruit	Untargeted	UPLC-MS/MS + NMR	Quality	Rivera-Mondragón et al., 2020
*Kigelia africana* (Lam.) Benth.	Fruit	Untargeted	UHPLC/GC-TOF-MS + GC–MS	Quality	Fagbohun et al., 2021
*Butia* spp. (Arecaceae)	Fruit	Untargeted	LC-MS	Environment	Hoffmann et al., 2017
Pear (*Pyrus bretschneideri* × *Pyrus pyrifolia*)	Pulp and pericarp	Untargeted	GC-MS + LC-MS	Environment	Rudell et al., 2017
Peach (*Prunus persica* L. Batsch)	Pulp and pericarp	Untargeted	GC-MS	Environment	Anthony et al., 2020
Apple *(Malus* × *domestica* Borkh*)*	Pulp and pericarp	Untargeted	NMR	Environment	D’Abrosca et al., 2017
Goji (*Lycium barbarum* L.)	Fruit	Untargeted	UPLC–ESI–MS/MS	Abiotic stress	Wei et al., 2020
Sweet cherry (*Prunus avium* L.)	Pulp and pericarp	Untargeted	GC–MS	Abiotic stress	Smith et al., 2011
Pomegranate (*Punica granatum* L.)	Stem and buds	Untargeted	HPLC-GC	Abiotic stress	Yazdanpanah et al., 2021
Sweet cherry (*Prunus avium* L.)	Fruit and stem	Untargeted	GC–MS	Quality	Karagiannis, 2018
Sweet cherry (*Prunus avium* L.)	Fruit	Targeted	HPLC	Quality	Fuentealba, 2021
Noni (*Morinda citrifolia* Linn.)	Pulp and pericarp	Untargeted	LC–MS	Quality	Wu et al., 2019
Noni (*Morinda citrifolia* Linn.)	Fruit	Untargeted	LC-MS	Quality	Wu et al., 2021
Mangosteen (*Garcinia mangostana* L.)	Pulp	Untargeted	GC–MS	Quality	Mamat et al., 2020
Pear *(Pyrus bretschneideri* × *Pyrus pyrifolia)*	Fruit	Targeted	HPLC	Quality	Lwin and Lee, 2020
Apple *(Malus* × *domestica* Borkh*)*	Pulp and pericarp	Targeted	GC	Quality	Rudell and Mattheis, 2009
Citrus (*Citrus reticulata*)	Pericarp	Untargeted	GC-TOF-MS	Quality	Fayek et al., 2019
Strawberry (*Fragaria* × *ananassa*)	Fruit	Untargeted	GC-MS + HPLC	Quality	Zhang et al., 2011
Starfruit (*Averrhoa Carambola* L.)	Fruit	Untargeted	HS-SPME-GC-MS	Quality	Ramadan et al., 2020
Mangosteen (*Garcinia mangostana* Linn.)	Fruit	Untargeted	GC-MS + LC-MS	Quality	R. Parijadi et al., 2019
Japanese persimmon (*Diospyros kaki* Thunb.)	Fruit	Untargeted	UPLC-MS	Quality	Ramírez-Briones et al., 2019
Japanese persimmon (*Diospyros kaki* Thunb.)	Fruit	Untargeted	NMR	Quality	Ryu et al., 2019
Mulberry fruit (*Morus alba Linnaeus*)	Fruit	Untargeted	GC-MS + HPLC	Quality	Lee et al., 2016
Grape berry (*Vitis vinifera* L.)	Fruit	Untargeted	LC-MS	Quality	Dai et al., 2013
Avocado (*Persea americana*)	Fruit	Untargeted	GC-APCI-TOF MS	Quality	Hurtado-Fernández et al., 2015

### Fruit Quality Formation

Fruit quality, such as fruit color, sweet/acid ratio, size, firmness, aroma, and special nutrients, is very important trait for consumers and is the main factor determining market competitiveness. Research on the mechanisms of fruit quality formation can reveal new information about fruit physiology, provide new biomarkers for variety or producing area identification, and accelerate the improvement of the fruit quality.

From a flower to a mature fruit, undergoing complex changes, metabolomics reveals the composition and changes of the compounds that form the fruit quality. A widely targeted metabolomic analysis in pineapple (*Ananas comosus* L.) young fruits (YF), mature fruits (MF), and fully mature fruits (FMF) identified 466 metabolites. In comparison, lysophospholipids (LPLs) were found to be the highest in YF, and their content decreased gradually with fruit development and remained almost constant in FMF (Hong et al., 2021). During the ripening of ginseng berry berries, the content of malate gradually increases, and most amino acids and organic acids gradually decrease. Compounds that could distinguish different cultivation ages and maturity stages of ginseng were obtained (Mamat et al., 2020). Content variation in amino acid and isoprenoid in *ASR1*-antisense transgenic tomato (*Solanum lycopersicum*) fruits and normal tomato was examined by targeted metabolomics. Amino acids and isoprenoids were found to be significantly different in these two species. The combined analysis with transcriptome data demonstrates that the Asr1 [i.e., abscisic acid (ABA), stress, and ripening 1] transcription factor is involved in the cascade regulation of red tomato fruit maturation (Dominguez et al., 2021). Special metabolites also accumulate in mangosteen (Mamat et al., 2020), ginseng berry (Park et al., 2019), starfruit (Ramadan et al., 2020), avocado (Hurtado-Fernández et al., 2015), strawberry (Zhang et al., 2011; Wang et al., 2021e), apple (Rudell and Mattheis, 2009; Risticevic et al., 2016, 2020; Jing and Malladi, 2020; Xu et al., 2020), grape berry (Dai et al., 2013), mulberry (Lee et al., 2016), cupressaceae (Falasca et al., 2014), olea (Olmo-García et al., 2018), walnuts (Wang et al., 2021b), and African baobab (Baky et al., 2021) during fruit maturing.

Fruit color not only affects appetite but also affects nutrients. By targeting carotenoids in white fleshed apricots (*Prunus armeniaca* L.) “Kuchebaixing” and orange fleshed apricots “Shushangganxing” at different developmental stages (i.e., S1–S4), 14 carotenoids and 27 carotenoid lipids were identified in apricot flesh. The comparison revealed significant differences in the carotenoid content between these two different apricot cultivars during S3 and S4 development and finally identified β-carotene and (E/Z)-octahydro lycopene as the key metabolites responsible for the differences in total carotenoid content in the different cultivars. Combination of metabolome and transcriptome reveals *PSY*, *NCED1*, and *CCD4* as key genes responsible for differential accumulation of the total carotenoid content in apricot fruit (Zhou et al., 2021). The red pericarp longan (*Dimocarpus longan* Lour.) (RP) and white longan (SX) were analyzed using widely targeted metabolomics; a total of 597 substances were identified in both white and red longan. Among them, 33 compounds, mainly flavonoids, were present in red-skinned longan and 23 compounds, mainly phenolic acids, were present in white-skinned longan. Cyanidin 3-*O*-glucoside, cyanidin 3-*O*-6″-malonyl-glucoside, and cyanidin *O*-syringic acid specially existed in RP longan. Delphinin 3-*O*-glucoside specially existed in SX longan. Accordingly, genes in the biosynthetic pathway of anthocyanin, such as *F3H*, *F3’H, UFGT, GST, MYB, bHLH, NAC, and MADS*, were detected significantly upregulated in RP longan using the transcriptome analysis (Yi et al., 2021). The analysis of six Tuscan sweet cherries (*Prunus avium* L.) endemic to Italy by non-targeted metabolomics successfully identified 15 metabolites in the positive ion mode and 14 metabolites in the anion mode. Most of these metabolites belong to flavonoids, including a highly potent antioxidant, cinchonain, while the highest flavonoid content was found in two varieties, namely, Crognola and Morellona (Berni et al., 2021). Anthocyanins also have important effects on fruit coloration in jujube (Zhang et al., 2020), fig (Wang et al., 2017), and *Cerasus humilis* (Ji et al., 2020).

The taste and aroma of the fruit are important intrinsic qualities of the fruit. A variation of metabolites in kiwifruit (*Actinidia chinensis*) flavor formation was revealed by widely targeted metabolomics to detect the content of sugar, organic matter, and volatile compounds in the flesh of kiwifruit during six developmental and six postharvest periods. A total of 34 flavor-related metabolites were identified. Further studies showed that the accumulation of metabolites in kiwifruit differed between the immature and mature stages. In the immature stage, the accumulated metabolites will tend to accumulate esters and sugars, and in the mature stage, they will tend to accumulate aldehydes, alcohols, ketones, and organic acids. In addition, a regulatory network that can regulate the production and accumulation of soluble sugars, organic acids, and volatiles was constructed by combining metabolomic and transcriptomic data analysis. This regulatory network can identify key structural and regulatory genes associated with flavor metabolism, key transcription factors that regulate the metabolism of soluble sugars and esters, and moreover, reveal their role in regulating the transcription of key structural genes involved in these metabolic pathways (Wang et al., 2022). Lipid compounds and phenylpropane derivatives were identified as the main components of the dates’ aroma by detecting 13 volatile compounds of dates (*Phoenix dactylifera* L.). In addition, 2,3-butanediol, hexaldehyde, hexol, and cinnamaldehyde can be used to distinguish between the different dates varieties (Khalil et al., 2017). Methyl hexatate, which was not detected in two other producing regions, is a volatile unique to *Averrhoa carambola* L. in Egypt (Ramadan et al., 2020). Special metabolites were differentially accumulated in different cultivar and wild persimmon varieties (Ramírez-Briones et al., 2019; Ryu et al., 2019), *Citrus* L. (Wang et al., 2016; Fayek et al., 2019; Villa-Ruano et al., 2019), and peach varieties (Karagiannis et al., 2021).

After the fruit is picked, the quality may change depending on the storage condition before entering the consumer’s table (Karagiannis, 2018; Wu et al., 2019, 2021; Lwin and Lee, 2020; Fuentealba, 2021). Comparing the changes of volatile compounds before and after 7 days of storage of 3 hairy peaches (*Prunus persica* L.) and 3 nectarines, it was found that the distribution of volatile compounds in fruits tended to be similar after storage. Using random forest analysis, it was possible to identify 15 volatile organic compounds, namely, 6 terpenes, 6 esters, 2 lactones, and 1 aromatic VOC, which differed between varieties before and after storage. Of which, 4 terpenes and 4 esters were positively correlated with nectarine. Another 16 VOCs highly correlated with 11 key VOC pathway genes (Muto et al., 2020). In ethephon-treated sweet cherries, fruit ripening and ethylene-related metabolites, such as malate and monosaccharides, have changed (Smith et al., 2011).

The development of metabolomics has made it possible to identify the active components of special fruit trees with medicinal value. Fifteen compounds were identified from the *Kigelia africana* fruit using UHPLC-TOF-MS and GC-MS. In which, physostigmine was demonstrated with an excellent anticancer activity (Fagbohun et al., 2021). In addition, nine antiaging compounds in *Nephelium lappaceum* (rambutan) seeds were identified (Lee et al., 2020). Genipap (*Genipa americana* L.) is a known fruit with medicinal value. Biomarkers after the administration of genipap in human urine is identified by LC-MS (Dickson et al., 2020).

### Biotic/Abiotic Stress on Fruit Tree

Biotic stress and abiotic stress are great challenges to the fruit tree with impact on production, quality, and survival. The biotic/abiotic stress causes physiological changes, which eventually causes corresponding metabolites changes. By metabolomics analysis and in comparison with normal fruit tree, differential metabolites will be identified as new biomarkers of the biotic/abiotic stress and serve for disease diagnosis. Key metabolites pathways responding to biotic/abiotic stress can reveal molecular mechanism about resistance to biotic/abiotic stress.

Several researches have been carried out to find biomarkers and pathology of biotic stress, especially in microbe infection. Huanglongbing (HLB) (*Candidatus Liberibacter* sp.) is a common disease on citrus. Infested sugar orange (*Citrus reticulata*) is divided into one normal type and three abnormal types based on fruit peel color. Non-targeted metabolomics was used, and 215 significant differences metabolites were found between abnormal and normal fruit. Two unique metabolites (i.e., *O*-caffeoyl maltotriose and prunetin) were detected only in normal pericarp. Besides, it is also revealed that “phenylpropanoid biosynthesis” pathway exhibited obvious enrichment in all comparison groups, according to the metabolic pathway enrichment analysis of differential metabolites (Wang et al., 2020). *Penicillium* spp. is a citrus fruits susceptible pathogen. The contents of sugars, organic acids, vitamin C, and D-citmonene decreased in the citrus peel after infection, and the amount of ethanol and beta-terpene alcohol increased (Tang et al., 2018). Amino acids and their derivatives were significantly higher in the citrus peel of roughing disorder (Lu et al., 2017). In mulberry and apple, pathogen infection altered the levels of abscisic acid and cytokinin (Gai et al., 2014; Klee et al., 2019). Bird foraging flower buds will affect the survival of fruit trees. Salvianolic acid A is more abundant in peach buds where birds are prone to forage and may be a potential metabolite that attracts birds to forage (Zhang et al., 2019).

The study on mechanism of resistance to disease is important for breed improvement. Scanning electron microscopy (SEM), transcriptome, and metabolomics analysis revealed that exogenous application of NaH_2_PO_4_2H_2_O (P) and abscisic acid (ABA) increased the expression of “Huangguan” pear (*Pyrus bretschneideri* × *Pyrus pyrifolia*) wax-related and calcium-regulated genes, as well as increased “Huangguan” pear resistance to brown spot disease during harvest and storage (Wang et al., 2021f). Widely targeted metabolomics analysis was used to analyze leaves of HLB-tolerant and -sensitive citrus (*Citrus reticulata*) varieties, covering primary and secondary metabolic pathways including carbohydrate metabolism, nucleotide metabolism, amino acid metabolism, energy metabolism, and biosynthesis of secondary metabolites. In which, aspartate and glutamate metabolism, purine metabolism, biosynthesis of plant hormones, and catabolic pathways were upregulated in the tolerant group. A total of 50 metabolites related to HLB tolerance were identified, and these metabolites were considered as potential markers for the identification of citrus HLB (Suh et al., 2021). It was found that phenylpropanes and their derivatives play an important role in citrus fruits to resistant *Penicillium* infection (Ballester et al., 2013). In addition, fruit trees also produce special metabolites to affect the microbial populations in the rhizosphere to help resist the invasion of pathogens (Padhi et al., 2019).

Improper fertilization and abnormal temperature are common abiotic stresses on fruit trees. Nitrogen fertilizer is often over-applied in apple (*Malus* × *domestica* Borkh) cultivation in China (Approx. 600–800 kg N ha^–1^). Although high nitrogen fertilizer application promoted apple yield and fruit weight per unit, at the same time, the carbon-to-nitrogen ratio, soluble sugars, flavonoids, and other fruit quality indicators decreased. The combined analysis of transcriptome, proteomics, and metabolomics revealed the global pattern of high nitrogen effect on apple fruits. High nitrogen significantly inhibited the accumulation of carbohydrates (sucrose, glucose, and alginose) and flavonoids (rutin, rhamnolipid-3-*O*-rutinoside, and trihydroxyisoflavone-7-*O*-galactoside) in the fruits. More carbon groups are used in the synthesis of amino acids (especially arginine) and their derivatives under high nitrogen conditions to enhance the nitrogen metabolism process (Wang et al., 2021a). Under low phosphorus stress, the content of flavonoids and anthocyanin in leaves increased, and the amino acid and their derivatives, organic acids, and flavonoids in roots increased. In contrast, under high phosphorus stress, a higher flavone content in the leaves and lower anthocyanins in the roots were found. Thus, apple seedlings respond to phosphorus stress by regulating the flavonoid pathway (Sun et al., 2021). Different concentrations of phosphate fertilizer were also found leading to changes in flavonoids in Chinese wolfberry (*Lycium barbarum* L.) (Wei et al., 2020).

### Influence of Environment on Fruit Tree

The growth and development of fruit trees need light, temperature, air, water, and other non-nature environment. The change of environment will affect the physiology of fruit tree and quality of fruit. Metabolomics provides new method and new insight to reveal the mechanism of environmental regulation on fruit tree.

Light plays an important role in fruit growth. Bagging apple fruit reduces insect infestation, but it also reduces light exposure and increases the risk of browning. As found by Wang et al. (2021c), the bagging resulted in the production of 50 differential metabolites, in which a significant decrease in flavonoids was positively correlated with peel browning. In general, the inner fruit of turmeric trees received less light. The LC-MS and GC-MS analysis showed that sucrose and sorbitol contents were higher in the outer fruit, while glucose and malic acid contents were higher in the inner fruit (Rudell et al., 2017).

Persistent low temperature favors fruit trees to break dormancy, and changing temperature has an adverse effect on germination. Research of flower buds of pears treated at different temperatures showed that metabolites associated with the pentose phosphate pathway, energy production, and the tricarboxylic acid cycle (TCA) cycle may have reduced the germination rate of pear buds (Horikoshi et al., 2018). In contrast, [H_2_CN_2_ (HC)] breaking of blueberry dormancy is associated with increases in soluble sugars, organic acids, and amino acids (aspartate, glutamate, and phenylalanine) (Wang et al., 2021d). Treated with low temperature after harvesting leads to decrease of xylose, galactose, galacturonic acid, glucuronate, glycine, and rhamnose (R. Parijadi et al., 2019). Studies on *Butia* spp. (Arecaceae) growing at different latitude, longitude, and altitude in Brazil have found that fruits at lower altitude are sweeter, and fruits at higher altitude contain more carotenoids and phenols (Hoffmann et al., 2017).

Metabolomics was used to analyze the variation in fruit quality of four main Spanish grape cultivars RJ43, CL306, VN31, and 1084 under simulated climatic conditions [i.e., expected temperature, CO_2_ content, and relative humidity (RH)] and different irrigation patterns (i.e., water sufficiency/water deficit) for 2,100 years. The results indicate that CO_2_ levels in 2100 will increase sugar accumulation and reduce acidity in the grapes, but this effect is partially mitigated by insufficient water. In addition, climate change and moisture conditions significantly affected the concentration and intensity of amino acid accumulation in grapes, but these effects were different in different varieties. Besides, CO_2_ content and water deficiency combined to reduce anthocyanin and anthocyanin/total soluble solids ratio in grapes. In general, although global climate change and moisture conditions can have a significant impact on grape quality, that is limited by the grape varieties. For example, RJ43 and CL306 are more affected by global changes, while 1,084 is relatively less affected (Arrizabalaga-Arriazu et al., 2021).

In addition, the biological environment can also affect the physiological and biochemical characteristics of fruit trees. Banana fruits near natural forests (near-NF) are very different from that distant from natural forests (distant-NF) in color, ripening characteristics, and susceptibility. Mature banana fruits harvested in near-NF contain more organic acids, GBAB (a four-carbon non-proteinogenic amino acid) and unsaturated fatty acids, and these metabolites increase the flavor and nutritional composition of the banana, while also enhancing the ability of the banana fruit to cope with biotic and abiotic stresses. Fruits harvested in distant-NF contain more glutamate and putrescine, which makes bananas have an unpleasant smell after harvest and is also bad for storage (Nascimento et al., 2019).

Rootstocks and interstocks are generally used to dwarf fruit trees to achieve light and simplified management of the orchard. Comparison of the metabolome of pears grafted to different stocks revealed a significant increase in the concentrations of flavonoids and phenolic acids in scions grafted to dwarf stocks, along with significantly decreased concentrations of D-sorbitol and D-mannitol in the roots. It indicates that flavonoids and phenolic acids are key compounds involved in reducing scion growth and dwarf rootstock may control tree growth by regulating carbohydrate partitioning from shoot to roots (Cui et al., 2021). Training can also have effects on apple tree metabolism (D’Abrosca et al., 2017; Anthony et al., 2020). Girdling improves the quality of the sweet cherry fruit. The anthocyanin content in the peel varied significantly between pruning systems, and the slender spindle training system was the most favorable for fruit growth (Michailidis et al., 2020).

### Genetic Mechanism of Fruit Tree

While the inheritance and variation of metabolites are controlled by smaller loci, traditional phenotypic traits are often located at larger loci. The addition of metabolomics can accelerate the research progress and candidate genes screening in fruit trees. By considering the metabolite levels as quantitative traits, the metabolic traits can be analyzed by QTL, i.e., metabolic QTL (mQTL) analysis (Carreno-Quintero et al., 2012). Combining metabolomics and GWAS, named mGWAS (Luo, 2015). Most of the mQTL work has been focused on *Arabidopsis thaliana*, *S. Lycopersicum* (Toubiana et al., 2015; Knoch et al., 2017), *Triticum aestivum*, *Oryza sativa*, and *Zea mays* (Gong et al., 2013; Jiang et al., 2013; Chen et al., 2021), and recently mQTL have also been applied in study the mechanism of fruit genetic basis.

To obtain hybrid plants with high-soluble solids content (SSC)/titratable acidity (TA) ratios in strawberries (*Fragaria* × *ananassa*), Vallarino et al. (2019) used GC-MS to detect primary metabolites in “232,” “1,392,” and their hybrid population F1 for 2 years. A total of 50 compounds were detected and successfully identified, including soluble sugars, organic acids, amino acids, soluble alcohols, etc. Pearson correlation analysis revealed possible synergistic regulation of fructose and glucose, glucose and fructose 6-phosphate, succinic acid and fumaric acid, and raffinose and succinic acid. The mQTL analysis was performed for these 50 major metabolites, SSC, TA, pH, and L-AA data using the constructed “232” × “1,392” linkage maps. The final results of 133 QTL localizations were obtained, and QTL for organic acids and sugars (glucuronide and succinate, raffinose and sucrose) were found in LG V-4. It was speculated that a common gene with pleiotropy for sucrose, raffinose, and succinate might cause these changes. The expression level of FvH4_5g03890 (glucose-6 -β 1-episomerase) gene was found to be negatively correlated with sucrose and raffinose contents by glucose biosynthesis, metabolism-related annotation and quantitative RT-PCR (Vallarino et al., 2019).

A hotspot of QTLs of phenolic compounds, including procyanidins and flavan-3-ols, in ripe apple fruits were mapped by mQTLs at the top of LG16 (Khan et al., 2012a). Higher expression of leucoanthocyanidin reductase gene (MdLAR1), which located on the LG16, was associated with a fourfold increase of procyanidin. Combining with expression analysis of structural and regulation genes of the phenylpropanoid and flavonoid pathways, MdLAR1 was identified the candidate gene for procyanidins and flavan-3-ols (Khan et al., 2012b).

A total of metabolic profiling of 101 F_2_-generation grape stems were analyzed by LC-HR MS, and 1,317 characteristic ions were detected. Among them, 19 were related to stilbene compounds, and 5 mQTLs were finally identified by QTL localization. Two large-effect mQTLs, corresponding to a stilbenoid dimer and a trimer, were jointly localized in the 25.0–27.8 Mbps of LG18. This range also included 48 genes of bio-resistance, 15 genes in flavonoid biosynthesis pathway, and 6 genes of triterpenoid biosynthesis. In addition, haplotype dosage effects of 3 mQTLs were statistically significant. Fruit trees with high quality and disease resistance can be screened simultaneously (Teh et al., 2019).

Two hundred and seven jujube varieties, including 25 wild and 182 cultivated jujubes (*Ziziphus jujuba* Mill.), were used to study domestication-driven changes of fruit bioactive molecules by a combination of genome-sequencing, widely targeted metabolomics, and mGWAS. Genome-sequencing revealed 109 domestication-relative sweeps, which mainly distributed on chromosomes 1, 4, and 10. A total of 2,985 metabolites were annotated by a metabolome library with various bioactive metabolites. In which, 187 metabolites were associated with domestication. Totally, 1,080 associated loci for 986 metabolites were identified by mGWAS. Among them, 15 triterpenoids, which were significantly reduced in cultivated jujube, existed at six major loci. Mutations in the promoter of an OSC gene associated with triterpenes content reduction during domestication. In addition, by comparing the metabolites of 24 dry and 31 fresh types of jujube, 203 significantly different metabolites were identified, of which 7 cyclopeptide alkaloids (CPA) were highly accumulated in the dry variety. An *N*-methyltransferase were identified the CPA candidate gene by divergence sweep identification and its genomic sequence variations were responsible for content of CPAs in 39 jujube accessions (Zhang et al., 2021).

## Perspectives

In recent years, fruit tree metabolomics-related research has increased rapidly, revealing the mechanism of fruit quality formation, biotic/abiotic stress, and environmental influence on fruit tree. Alternatively, the obtained differential metabolites can be used as novel biomarkers for the identification of variety, origin, and pathogen infection. Metabolomics-transcriptomic combined analysis, further revealed the gene/metabolic regulatory network and candidate genes leading to trait. Metabolomics, combining with GWAS/QTL, narrows the range of candidate genes and increases the odds of acquiring key genes.

However, the development of metabolomics in fruit trees is still in its initial stage, and most studies are the method exploration and simple repetition of metabolomics application to fruit trees, obtaining differential metabolites but lacking in-depth mechanistic research. If the changes in the metabolome are combined with the changes in the organ, tissue, cellular, and molecular levels, the mechanism of trait changes should be even more systematically revealed. In addition, non-targeted metabolome is widely used in fruit tree metabolomics research, but only a few dozen compounds in thousands obtained can be identified, which not only leads to low metabolomics efficiency, but also the differential metabolites obtained may not real represent differences. The current metabolic library is mainly prepared for human, animal, and drug experiments and rarely covers the unique metabolites of fruit trees. The construction of metabolomics database of fruit tree will greatly promote the application of metabolomics in fruit tree. Widely targeted and pseudo-targeted metabolomes allow the quantitative and qualitative acquisition of thousands of compounds and are alternative solutions if the cost is reduced. Then, extreme metabolite diversity not only makes the fruit tree metabolomics study particularly required but also brings challenges to the various stages of metabolomics experiments, analysis, and library building. To further promote the application of metabolomics to fruit trees, the following problems or bottlenecks should be solved. Firstly, a more broad-spectrum and universal detection method should to be constructed. Secondly, a really unbiased, high-sensitive, and high-throughput metabolism analysis platform with the ability to parallel analyze huge amounts of data is needed. Thirdly, accurate and efficient identification or annotation of metabolites database or tools should be developed.

With the improvement of instruments and metabolite database and decrease of cost of experiment, metabolomics will prompt the fruit tree research to achieve more breakthrough results.

## Author Contributions

JL, ST, GY, XD, XH, and JW provided suggestions for the frame structure and content of the manuscript. JL wrote the first draft. ST assisted in the translation of the manuscript. GY and XD provided help in the arrangement of the table. KZ, GY, XD, XMZ, YZ, CW, and XZ contributed to the revision of the manuscript. All authors contributed to the article and approved the submitted version.

## Conflict of Interest

The authors declare that the research was conducted in the absence of any commercial or financial relationships that could be construed as a potential conflict of interest.

## Publisher’s Note

All claims expressed in this article are solely those of the authors and do not necessarily represent those of their affiliated organizations, or those of the publisher, the editors and the reviewers. Any product that may be evaluated in this article, or claim that may be made by its manufacturer, is not guaranteed or endorsed by the publisher.

## Footnotes

1
https://www.webofscience.com/
