# Corrigendum: Research progress and trends in metabolomics of fruit trees

**DOI:** 10.3389/fpls.2023.1192425

**Published:** 2023-04-05

**Authors:** Jing Li, Guohua Yan, Xuwei Duan, Kaichun Zhang, Xiaoming Zhang, Yu Zhou, Chuanbao Wu, Xin Zhang, Shengnan Tan, Xin Hua, Jing Wang

**Affiliations:** ^1^ Key Laboratory of Saline-Alkali Vegetation Ecology Restoration, Ministry of Education, Northeast Forestry University, Harbin, China; ^2^ Institute of Forestry and Pomology, Beijing Academy of Agriculture and Forestry Sciences, Beijing, China; ^3^ Key Laboratory of Biology and Genetic Improvement of Horticultural Crops (North China), Ministry of Agriculture and Rural Affairs, Beijing, China; ^4^ Beijing Engineering Research Center for Deciduous Fruit Trees, Beijing, China; ^5^ Analysis and Test Center, Northeast Forestry University, Harbin, China

**Keywords:** fruit tree, metabolomics, quality, resistance, mQTL, mGWAS

In the published article, there was an error in the Funding statement. There were two funds not included. They are the “Youth Research Fund of the Beijing Academy of Forestry and Fruit Sciences (LGYJJ202006, LGYJJ202005)”. The correct Funding statement appears below.

